# Oxidative stress and type 2 diabetes: the development and the pathogenesis, Jordanian cross-sectional study

**DOI:** 10.1186/s40001-024-01906-4

**Published:** 2024-07-17

**Authors:** Khalid M Abu Khadra, Mohammad Izzat. Bataineh, Ahmad Khalil, Jumana Saleh

**Affiliations:** 1 Department of Biological Sciences, Yarmouk University, Irbid, 21163 Jordan; 2 Biochemistry Department, College of Medicine and Health Sciences, Sultan Qaboos University, 123 Muscat, Oman

**Keywords:** Catalase, Malondialdehyde (MDA), Diabetes, Obesity, Oxidative stress, Antioxidant

## Abstract

Accumulation of reactive oxygen species (ROS) can disrupt the antioxidant defense system, leading to oxidative stress that leads to pathological damage to vital human organs, including hormone-producing glands. Normal physiological function is subsequently disrupted and disorders such as Type 2 Diabetes Mellitus (T2DM) may develop. The critical role of the antioxidant defense system in counteracting ROS and mitigating oxidative stress is fundamental to understanding the pathogenesis of T2DM. In our study, we monitored the oxidant/antioxidant status in a selected Jordanian population to further elucidate this relationship. Our results show higher serum levels of Malondialdehyde (MDA); 0.230 ± 0.05 and 0.207 ± 0.06 μmol/l for the diabetic and the obese groups, respectively, relative to 0.135 ± 0.04 μmol/l for the non-obese healthy group. Lower activity of Catalase (CAT) was recorded among the diabetic (9.2 ± 3.2) and obese groups (11.0 ± 2.8), compared to the non-obese healthy group (12.1 ± 3.5). Significant elevations (*P* < 0.05) were observed in uric acid concentrations in diabetic and obese subjects: 451 ± 57 mg/dl and 430 ± 51, respectively, versus 342 ± 57 mg/dl in the non-obese healthy group. Moreover, no significant differences were obtained between all the studied groups for the serum albumin and total protein concentrations. Our findings demonstrate the potential role of oxidative stress in the development and occurrence of T2DM.

## Background

Disturbance in the antioxidant system or the imbalance between the production of reactive oxygen species (ROS) and antioxidant capacity could cause human tissue damage and create pathological changes [1, 2]. The overproduction and accumulation of ROS may result in oxidative stress [3, 4]. Oxidative stress is a condition where ROS interacts with cellular biological molecules causing irreversible tissue damage in human organs such as the kidneys, heart, as well as hormone-producing glands [5, 6]. Vital organ tissue damages rattle’s normal physiological function and subsequently develops a disease with chronic consequences [1].

Oxidative stress imposes a harmful effect on cells and tissues and is found to be associated with diabetic hyperglycemia after interacting with cellular biomolecules including proteins and lipids [7, 8]. One prominent harmful consequence of ROS and oxidative stress is lipid peroxidation which deteriorates cellular structure and function [6]. If the production of ROS exceeds the antioxidant capacity of the cell, oxidative stress develops leading to lipid peroxidation [9, 10]. The final product of lipid peroxidation is malondialdehyde (MDA) which mediates cell membrane damage and is a prominent biomarker of lipid peroxidation [11, 12]. The conversion of superoxide anion radicals (O2⋅−) to hydrogen peroxide and oxygen is catalyzed by superoxide dismutase (SOD) followed by catalase (CAT). These events point out that CAT and SOD are cellular antioxidant enzymes that counter oxidative stress [13, 14]. Accordingly, oxidative stress created after free radical overproduction provokes antioxidant enzymes [1, 2].

Research studies found that oxidative stress and obesity are associated with Type 2 Diabetes Mellitus (T2DM) [15, 16]. Numerous studies have attempted to determine whether oxidative stress is a causative factor in Type 2 Diabetes Mellitus (T2DM) due to hyperglycemia, or if it is a consequence that exacerbates dysfunction in the pancreas and insulin-associated receptors, ultimately contributing to the development of T2DM [17].

In this regard, investigating oxidative stress-related enzymes and antioxidant activities among T2DM patients and obese individuals could probe whether oxidative stress is an etiology or a complication [18]. Diabetes mellitus (DM) is a universal public health issue and the seventh primary cause of death worldwide. In Middle Eastern and North African countries, DM prevalence ranks second highest worldwide [19]. In Jordan, the T2DM epidemic is expected to grow in the following three decades, propelled by high and increasing obesity levels as well as an aging population [20]. Diabetes is a metabolic disorder of multiple etiological factors that occurs mainly due to impaired secretion of insulin by damaged pancreatic beta cells or impaired insulin activity leading to failure in the metabolism of carbohydrates, proteins, and lipids [21]. The association between T2DM and oxidative stress was demonstrated by elevated MDA levels and the activities of SOD and CAT [22, 23]. Moreover, an association between oxidative stress and T2DM complications was established [15]. Evaluating and comparing antioxidant enzymes among T2DM patients and obese individuals will help in resolving the role of oxidative stress and antioxidant capacity in the development of T2DM particularly in obese individuals. Therefore, we designed this study to investigate the association between oxidative stress and the occurrence of T2DM based on the analysis of oxidative stress biomarkers shared with obese individuals. This study analyzed the antioxidant profile of selected Jordanian T2DM patients and obese healthy volunteers separately by measuring MDA levels, CAT activity, albumin, uric acid, and serum total proteins aiming to explore and illuminate their potential implications.

## Materials and methods

### Ethical considerations

All the protocols described herein were approved by the administration of The Princess Basma Teaching Hospital, Irbid, Jordan, and the Institutional Review Board Council at Yarmouk University. Participants who volunteered to participate were informed about the study’s goal and significance. Participants’ rights for confidentiality, privacy, and safety were assured. Participants were informed that the collected information would only be for research purposes, and not to be accessed by anyone except the researchers. The eligible patients signed a free and informed consent form. The T2DM patients were chosen and recruited from patients who were referred to the diabetic care clinic. Diabetes was defined according to the American Diabetes Association (ADA), diagnostic criteria, fasting plasma glucose > 126 mg/dl, HbA1c ≥ 6.5%, and receiving diabetes treatment [24]. The control was randomly recruited from volunteers from the Jordanian community and the inclusion criteria for the control participants were having no history of glucose intolerance, and fasting plasma glucose less than 100 mg/dl. Any patient who is a smoker, pregnant, taking antioxidant drugs, or has been exposed to X-rays in the last three months was excluded from this study. All questions by the participants were answered honestly and completely. Direct interviews were conducted with each participant by a post-graduate student trained for this purpose.

### Subjects

This prospective cross-sectional study recruited 406 adults [186 (45.8%) males and 220 (54.2%) females] from April to June 2018, aged between 35 and 53 years. The sample included 201 T2DM patients [101 (50.2%) males and 100 (49.8%) females], and 205 non-T2DM groups [85 (41.5%) males and 120 (58.5%) females]. The participants in the non-T2DM group were divided into two groups, obese and non-obese. This classification was based on the calculated body mass index (BMI) after measuring the weight and the height and dividing the weight (kg) by the height squared (m). Obesity was determined according to the WHO definition of obesity. The BMI for the non-obese group was from 20 to 29.9 kg/m^2^, and the BMI for the recruited obese participants was from 30 to above 40 kg/m^2^.

### Biochemical testing

Peripheral blood samples (10 ml) were obtained from each person in a plain tube. The participant was asked to fast for 12 h proceeding the time of blood collection. The collected blood was centrifuged at 5000 rpm for 10 min at 4 °C to obtain serum. Obtained serum was utilized to measure the specified oxidative stress parameters and antioxidant enzymes counting uric acid, total protein, albumin, CAT, and MDA levels. Serum glucose concentration was measured based on the conventional hexokinase method. Uric acid, total protein, and albumin serum levels were determined using conventional specific colorimetric assays for each test. MDA serum levels were determined using a quantitative colorimetric method. The method was based on the reaction of MDA with thiobarbituric acid (TBA**)** [25]. The MDA assay was performed by taking 40 μl serum, 160 μl of phosphate buffer, 10 μl of 24 synthetic antioxidants (BHT), and 100 μl of 30% TCA. After mixing for a short time, the tubes were incubated at − 20 °C for 2 h and centrifuged at 2000 rpm for 15 min. The supernatant (1 ml) was transferred to another Eppendorf tube. Then, 20 μl EDTA-Na2H2O and 50 μl TBA were added to the supernatant and incubated in a water bath (90 °C) for 15 min with gentle vortexing. The absorbance of the pink-colored complex formed by the MDA-TBA adduct was measured at 532 nm to quantify the MDA using a UV–Vis spectrophotometer (BEL Engineering SRL, Monza (MB) Italy). The activity of CAT was determined according to the method followed by Aebi et al. [26]. Briefly, 100 μl of serum was mixed with an equal volume of absolute alcohol. The mixture was incubated for 8 min in an ice bath for the degradation of inactive CAT and the release of active CAT enzyme. The ice-incubated tube was brought back to room temperature before the addition of 10 μl of Triton X-100. The sample mixture (50 μl) was added to 200 μl of phosphate buffer and 250 μl of 0.066 M H_2_O_2_ as substrate. This was followed by mixing thoroughly before measuring the decrease in absorbance at 240 nm for 30 s. A molar absorptivity of 43.6 M cm^–1^ was used to calculate CAT activity, one unit of which is equal to μM of H_2_O_2_ degraded per min per mg of protein.

### Statistical analysis

Data for the three matched groups were compared and data from biochemical analyses were subjected to analysis of variance (ANOVA) by one-way analysis of variance (one-way ANOVA), using SPSS 17 (Statistical Package for Social Sciences Program, version 17, Chicago, USA) [27]. Means were compared by using Duncan tests at alpha = 0.05. A probability of *P* < 0.05 was considered a significant difference.

## Results

This study recruited 406 adults (186 (45.8%) males and 220 (54.2%) females) aged between 35 and 53 years. The diabetic group included 201 T2DM patients (101 (50.2%) males and 100 (49.8%) females). The obese non-diabetic group included 90 individuals (35 (39%) males and 55 (61%) females). The non-obese healthy control group included 115 individuals (50 (43.5%) males and 65 (56.5%) females), Table 1.

**Table 1:** Anthropometric and biochemical parameters (*n* = 406)

Variable	T2DM (*n* = 201)	Non-diabetic obese (*n* = 90)	Non-obese (healthy group) (*n* = 115)
Age (Y)	49.0 ± 4.6	45.9 ± 5.4	41.7 ± 6.0
Male	101	35	50
Female	100	55	65
BMI	31.4 ± 5	34 ± 3.9	25.3 ± 2.7
UA (μmol/l)	451 ± 57	430 ± 51	342 ± 57*
FBS (mmol/l)	10.0 ± 4.5	5.5 ± 0.65	5.2 ± 0.53
HbA1c (%)	7.85 ± 1.8	5.40 ± 0.42	5.2 ± 0.37
Albumin (g/l)	41.5 ± 5.8	42.3 ± 4.7	41.8 ± 6.0
Total Protein (g/l)	71.5 ± 5.4	71.8 ± 4.5	71.6 ± 4.5

* Significantly lower than obese and diabetic P< 0.05

Data are presented as mean ± SD

**Fig. 1:**
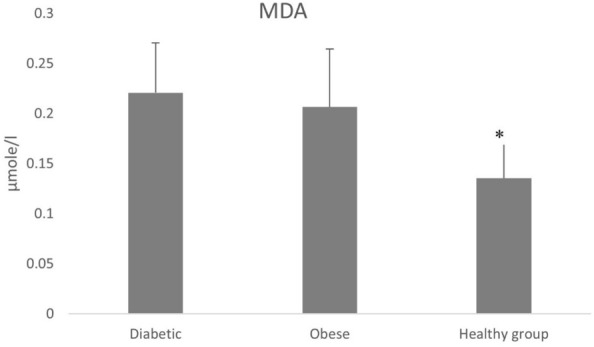
The mean MDA levels in the blood (µmole/l), for diabetic (T2DM), obese, and non-obese healthy groups. There was a significant difference between the non-obese healthy group, and diabetic, and obese groups, respectively (*P* < 0.05); no significant difference was found between the diabetic and obese groups

**Fig. 2:**
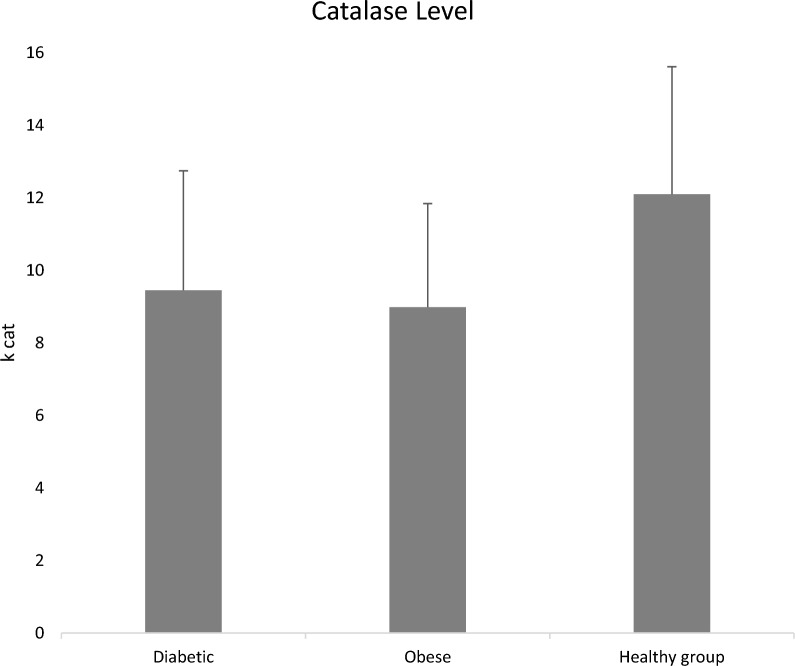
The mean catalase activities (*k*_cat_), for the diabetic, obese, and non-obese healthy groups, show a significant difference between the non-obese healthy group, and diabetic, and the obese group, respectively (*P* < 0.05)

Higher MDA levels were found for the diabetic group (0.230 ± 0.05) and the obese group compared to the non-obese healthy group (*P* < 0.05) (Fig. 1). Our results showed a significant difference in MDA levels between the obese group and the diabetic group (*P* < 0.05). The mean serum CAT activity for the diabetic group and the obese group was significantly lower than that of the non-obese healthy group (*P* < 0.05) (Fig. 2). However, there was no significant difference between the diabetic group and the obese group in CAT activity (Fig. 2).

Regarding uric acid, the results demonstrated that diabetic individuals showed significant elevations in uric acid concentrations compared with the non-obese healthy group (*P* < 0.05) (Table 1). On the other hand, the mean uric acid concentrations for the non-obese healthy group were much lower than that obtained for the obese group (*P* < 0.05) (Table 1). The differences in uric acid levels between the diabetic and obese groups were not significant (*P* < 0.05).

The mean albumin concentration among the diabetic group was comparable to that of the non-obese healthy group (Table 1). Similarly, the differences between the mean albumin concentrations for the obese group and the non-obese healthy group were not significant (*P* ˃ 0.05) (Table 1). No significant differences (*P* ˃ 0.05) were found for total protein concentrations among the three study groups (Table 1).

## Discussion

Recent research reveals that oxidative stress plays a critical role in the development of various metabolic disorders including T2DM, [1, 17]. Research findings regarding the association between oxidative stress and T2DM were based on the development of oxidative stress because of diabetic hyperglycemia. Herein, we aimed to investigate the hypothesis that may establish a groundwork for potential mechanisms for oxidative stress as a significant etiological factor in the initiation, and progression of diabetes.

Obesity was claimed to be involved in the development of T2DM through insulin resistance [28]. The associations between obesity, insulin resistance, and development of T2DM have been studied suggesting several hypothesized mechanisms including oxidative stress [15, 29, 30]. The biomarkers of oxidative damage are higher in individuals with obesity and correlate directly with BMI [16, 31]. Previous research showed that a diet high in fat and carbohydrates enhances a significant increase in oxidative stress and inflammation in persons with obesity [32]. Several studies have reported an association between obesity and oxidative stress through the irregular production of factors called adipokines [33]. Accordingly, obesity could have a role in increasing oxidative stress and its metabolic complications. Matsuda and Shimomura [15] showed increased levels of oxidative stress among obese non-diabetic individuals. Our postulated hypothesis regarding oxidative stress as an etiological factor for T2DM is based on the increased levels of oxidative stress parameters and declines in antioxidant defense measures among both obese and diabetic patients concurrently. Our results revealed significantly higher MDA levels in diabetic and obese individuals compared to the non-diabetic non-obese groups. These results were consistent with the previous findings of similar studies analyzing the association between obesity and oxidative stress [34, 35]. Moreover, an increase in the MDA levels in association with increased BMI was obtained indicating that obesity could predispose to free radical-mediated lipid peroxidation [10].

The results of the current research support data published earlier regarding catalase activity in diabetic people [3]. Significantly lower measures of CAT activity were noted among the diabetic and obese groups compared to non-obese healthy groups. Consequently, low CAT activity among obese and diabetic groups compared to healthy groups supports the notion that oxidative stress and defects in antioxidant enzyme function may be a predisposing factor in obesity that leads to the development of T2DM. Uric acid acts as an effective eliminator of free radicals and is classified as a scavenger of ROS including single oxygen and hydroxyl radicals [36]. As an effective eliminator of free radicals, uric acid has a crucial role as a metabolite by-product diminishing oxidative stress [1, 37]. We observed that uric acid levels were significantly higher among obese individuals and diabetic patients compared to non-obese healthy subjects. These results are compatible with reported data showing a positive association between elevated serum uric acid levels and diabetes [38, 39], and contradict earlier investigations reporting no positive association between serum uric acid and T2DM [40, 41]. Also, they do not agree with the data indicating an inverse relationship between serum uric acid levels and T2DM [42]. Our results show no difference in uric acid levels between diabetic and obese groups, but both groups had higher levels than non-obese healthy groups, suggesting that obesity and diabetes have the same effect on uric acid levels. These findings are in harmony with the results of Fabbrini et al. [43], who suggested that uric acid is an important antioxidant element and might prevent damage resulting from oxidative stress in obese individuals. Concerning albumin levels, the present findings did not show any significant difference between all groups, even though albumin has an antioxidant defense function and plays a role in the elimination of reactive nitrogen and oxygen species [44]. A previous study revealed a significant increase in albumin levels among individuals suffering from both types of diabetes [45]. Total protein levels among the diabetic group and the two healthy obese and non-obese control groups were near the total protein levels obtained from other studies that analyzed total protein levels in diabetic and non-diabetic groups. Our research offers significant insights into the potential role of oxidative stress in the development of T2DM. This assertion is supported by marked changes in oxidative stress indicators observed for both diabetic and obese individuals. Our findings suggest that obesity may play an independent role in alterations in oxidative stress and antioxidant measures, contributing to the onset and progression of diabetes. This study supports the hypothesis that positions oxidative stress as a potential etiological factor and highlights obesity as an emerging mechanistic link to the development of diabetes. Elevated levels of oxidative stress biomarkers in individuals with obesity align with existing research, emphasizing the association between oxidative stress and T2DM. Furthermore, the observed lower activity of the antioxidant enzyme catalase in diabetic individuals underscores the potential role of oxidative stress and antioxidant enzyme defects in T2DM. Surprisingly, elevated uric acid levels in both obese individuals and diabetic patients challenge conventional perspectives, suggesting a potential protective role against oxidative stress. This finding prompts a re-evaluation of the relationship between serum uric acid and T2DM, contrary to some previous studies. However, no significant differences in albumin levels were observed across groups, despite its known antioxidant function.

Overall, there is no doubt that the relationship between oxidative stress and T2DM is complex, with many dynamics yet to be fully elucidated. Considering the gap in the literature regarding the role of oxidative stress as a causative factor or a consequence of T2DM, our study provides direction toward the narrative that oxidative stress, associated with weight gain, may predispose T2DM development, and suggests that oxidative stress is not solely a consequence of diabetes but may rather be a triggering factor. Therefore, weight gain-induced oxidative stress may play a pivotal role in the pathogenesis of insulin resistance and subsequent development of T2DM. Therefore, while acknowledging the existing gaps regarding oxidative stress in T2DM, our research provides insights into the intricate interplay between systemic changes, weight gain, and oxidative stress. These insights may hold profound implications for understanding and managing the pathogenesis of diabetes.

In conclusion, our study provokes interest in considering an interplay between oxidative stress, obesity, and T2DM. These insights not only deepen our knowledge of underlying mechanisms but also offer avenues for targeted therapeutic interventions and lifestyle modifications to mitigate T2DM-related risks and complications. While our findings are valuable, it is essential to acknowledge certain limitations, such as the cross-sectional design, which may not reflect dynamic changes over time, and the potential influence of unmeasured factors. Further limitations include the regional scope of the study which may be affected by cultural and environmental factors. Future research, including regional and international association studies and interventional designs, will be crucial to address these gaps and establish causative mechanisms and disease predictors at a wider scale. However, the prospective nature of our study design ensures consistency and reliability, offering a snapshot of the current state of the study population and identifying emerging trends or developments.

## Data Availability

Patient-level data will be available on request from the corresponding author.
